# Multicenter randomized controlled trial and registry study to assess the safety and efficacy of the NanoKnife® system for the ablation of stage 3 pancreatic adenocarcinoma: overview of study protocols

**DOI:** 10.1186/s12885-021-08474-4

**Published:** 2021-07-07

**Authors:** Govindarajan Narayanan, Malcolm M. Bilimoria, Peter J. Hosein, Zhaohui Su, Kathleen M. Mortimer, Robert C. G. Martin

**Affiliations:** 1 Interventional Oncology, Miami Cancer Institute, Baptist Health of South Florida, Miami, FL USA; 2 Northwest Community Health, Arlington Heights, IL USA; 3 Department of Medicine, University of Miami Miller School of Medicine, Miami, FL USA; 4 OM1, Inc., Boston, MA USA; 5 Department of Surgery, University of Louisville, Louisville, KY USA

**Keywords:** Pancreatic ductal adenocarcinoma, Locally advanced pancreatic cancer, Irreversible electroporation, NanoKnife system, Ablation, Modified FOLFIRINOX

## Abstract

**Background:**

Irreversible electroporation (IRE) is a local ablation technique utilizing high voltage, low energy direct current to create nanopores in cell membrane which disrupt homeostasis and leads to cell death. Previous reports have suggested IRE may have a role in treating borderline resectable and unresectable Stage 3 pancreatic tumors.

**Methods:**

Patients with Stage 3 pancreatic ductal adenocarcinoma (PDAC) will be enrolled in either a randomized, controlled, multicenter trial (RCT) or a multicenter registry study. Subjects enrolled in the RCT must have no evidence of disease progression after 3 months of modified FOLFIRINOX (mFOLFIRINOX) treatment prior to being randomization to either a control or IRE arm. Post-induction and post-IRE treatment for the control and IRE arms, respectively, will be left to the discretion of the treating physician. The RCT will enroll 528 subjects with 264 per arm and include up to 15 sites. All subjects will be followed for at least 24 months or until death. The registry study will include two cohorts of patients with Stage 3 PDAC, patients who received institutional standard of care (SOC) alone and those treated with IRE in addition to SOC. Both cohorts will be required to have undergone at least 3 months of SOC without progression prior to enrollment. The registry study will enroll 532 patients with 266 patients in each arm. All patients will be followed for at least 24 months or until death. The primary efficacy endpoint for both studies will be overall survival (OS). Co-primary safety endpoints will be 1) time from randomization or enrollment in the registry to death or new onset of Grade 4 adverse event (AE), and (2 high-grade complications defined as any AE or serious AE (SAE) with a CTCAE v5.0 grade of 3 or higher. Secondary endpoints will include progression-free survival, cancer-related pain, quality of life, and procedure-related pain for the IRE arm only.

**Discussion:**

These studies are intended to provide Level 1 clinical evidence and real-world data demonstrating the clinical utility and safety of the use of IRE in combination with chemotherapy in patients with Stage 3 PDAC.

**Trial registration:**

Clinicaltrials.gov NCT03899636 and NCT03899649. Registered April 2, 2019. Food and Drug Administration (FDA) Investigational Device Exemption (IDE) trial G180278 approved on May 3, 2019.

## Background

The incidence of pancreatic cancer has risen consistently from 1992 to 2020, while the number of deaths due to pancreatic cancer in the United States has also risen proportionately [1]. There will be an estimated 57,600 patients initially diagnosed pancreatic cancer in 2020, representing 3.2% of all new cancer cases. A total of 47,050 deaths due to pancreatic cancer are projected to occur in the U.S. in 2020 representing 7.8% of all cancer deaths [1]. Median overall 5-year survival for patients with Stage 1 and 2 pancreatic cancer has been reported to be 24.4 months while locally advanced pancreatic Stage 3 cancer (LAPC) has a median survival of less 1 year [2, 3]. Patients with Stage 3 pancreatic ductal adenocarcinoma (PDAC) represent 39.2% of the nonmetastatic patient population with an associated 5-year survival of 10.8% [2]. The probability of survival is inversely proportional to tumor size and the number of positive lymph nodes [4].

The current standard of care (SOC) for stage 3 PDAC includes systemic chemotherapy. FOLFIRINOX (combination chemotherapy using 5-fluorouracil [5-FU], leucovorin [folinic acid], irinotecan, and oxaliplatin) has demonstrated improved overall survival to 11.1 months for FOLFIRINOX versus 6.4 months for gemcitabine in metastatic pancreatic cancer, but at the cost of greater toxicity [5]. More recently, a modified form of FOLFIRINOX (without the bolus 5-FU and with a reduced dose of irinotecan) has been shown to have an acceptable safety profile while maintaining comparable efficacy of FOLFIRINOX in metastatic pancreatic cancer [6]. The National Cooperative Cancer Network (NCCN) has recently added modified FOLFIRINOX (mFOLFIRINOX) as a preferred regimen for Stage 3 pancreatic cancer patients with a good performance status (ECOG 0–1) [7].

Although radiation therapy is frequently utilized in the United States for patients with Stage 3 pancreatic cancer, there currently exists a dearth of level 1 evidence demonstrating benefit for radiation or any other local therapy modality. One of the pivotal studies for radiation therapy in this population was the LAP07 prospective randomized trial [8]. In this trial, patients received induction chemotherapy with gemcitabine with or without erlotinib and were then randomized to receive chemoradiation therapy or continue chemotherapy. There was no difference in the primary endpoint of OS although there was decreased local tumor progression in the chemoradiation arm. One major limitation of this trial is that the induction chemotherapy used was not consistent with the current standard of using multiagent combination chemotherapy and it is possible that a more effective systemic induction regimen like mFOLFIRINOX may set the stage for benefit from a local intervention like radiation or ablation.

The poor outcomes among patients with pancreatic cancer has led to the pursuit of new treatment options. Irreversible electroporation or IRE (NanoKnife System, AngioDynamics, Inc., Latham, New York) is a non-thermal based method for local ablation which causes increased permeabilization of the cell membrane through exposure of the cell to electric pulses [9]. Electrodes are placed in a pattern which enables the tumor to be encompassed by the electrical field produced, with electric pulses irreversibly permeating the membranes resulting in cell death. IRE may lead to better preservation of vessels, nerves, and extracellular matrix within or close to the ablated area compared to thermal ablation techniques [10–13].

A growing body of evidence exists on the use of the NanoKnife System to treat pancreatic tumors, including both borderline resectable and unresectable tumors [14–16]. Studies have reported a median OS from diagnosis ranging from 14 to 27 months [17–27] and a median OS from IRE treatment ranging from 10 to 27 months [28–30]. The largest of these studies, a 200-patient, multicenter registry of patients with stage 3 pancreatic cancer treated with IRE via an open surgical approach, reported a median OS of 24.9 months and a median local progression free interval of 10.7 months [21]. Holland et al. recently reported results from a multicenter registry which included 152 patients with LAPC treated with IRE via an open approach at 6 different institutions [31]. Median OS from diagnosis, progression-free survival (PFS), and time to progression (TTP), all from diagnosis were 30.7 months, 22.8 months, and 27.3 months, respectively.

Narayanan et al. reported a retrospective review of 50 patients with LAPC treated with percutaneous IRE (26). Median OS was 27 months (95% confidence interval [CI], 22.7–32.5 months from the time of diagnosis) and 14.2 months (95% CI, 9.7–16.2 months) from the time of IRE. Multivariate analysis demonstrated patients with tumors ≤3 cm had significantly longer median OS than patients with tumors > 3 cm (33.8 vs. 22.7 months from time of diagnosis; *p* = 0.002, and 16.2 vs. 9.9 months from time of IRE; *p* = 0.031). More recently, Ruarus et al. reported the results of the Phase II multicenter PANFIRE II study which prospectively enrolled 50 patients treated with CT-guided percutaneous IRE [25]. This included 40 patients with LAPC and 10 patients with local recurring pancreatic cancer following resection. The median OS from diagnosis for patients with LAPC was 17 months. For patients with local recurrence, the median OS was 16 months from the diagnosis of recurrence and 9 months from IRE treatment. He et al. retrospectively compared 36 patients treated with IRE and 40 patients receiving radiotherapy with both groups receiving 4 months of induction chemotherapy prior to treatment [32]. Following a Propensity Score Matching (PSM) analysis, patients in the IRE group had longer median OS than patients receiving radiotherapy (21.6 months vs. 10.6 months) with significantly greater 1-year (71.4% vs. 41.3%) and 2 year (53.5% vs. 20.7%) OS rates (*p* = 0.011).

Major complications (grade 3 or higher) have been reported in approximately 21 to 34% of patients treated with IRE [16, 25]. This includes the development of portal vein thrombosis, pancreatic fistulae, pancreatitis and hematomas [15].

The above studies have paved the way for conducting two U.S. Food and Drug Administration (FDA) approved Investigational Device Exemption (IDE) studies to prospectively investigate the safety and efficacy of IRE treatment in patients with Stage 3 PDAC, with the goal of establishing level 1 clinical evidence via a RCT and the collection of real-world data via a parallel registry study that facilitates the enrollment of a similar, yet broader, patient population.

## Methods

### Randomized controlled trial (RCT)

**Fig. 1:**
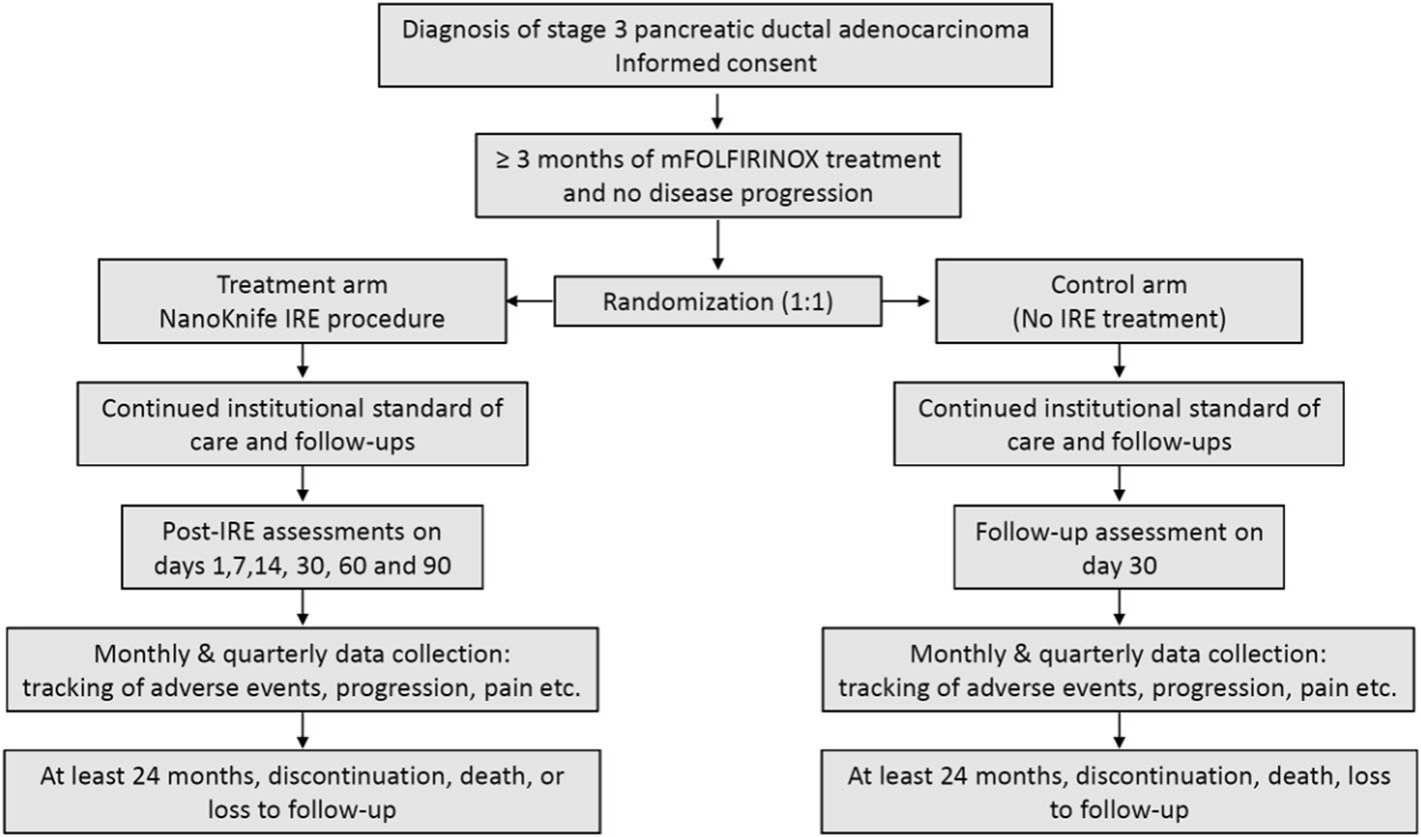
Study Design: Randomized Controlled

The randomized, controlled, 2-arm, unblinded multicenter trial will be conducted at up to 15 sites. After subjects have been treated with 3 months of mFOLFIRINOX and there is no evidence of disease progression, they will be randomized to either a control arm or a treatment arm of IRE with the NanoKnife System using either an open or a percutaneous approach. Randomization will be conducted centrally with subjects randomized in a 1:1 ratio after assessment using a study specified imaging protocol. (Fig. 1). Post-induction treatment in the control arm and post IRE treatment for the IRE arm will be left to the discretion of the treating physician. The mFOLFIRINOX regimen will consist of oxaliplatin at 85 mg/m2, leucovorin at 400 mg/m2, irinotecan at 150 mg/m2 all on day 1, plus 5-FU at 2.4 g/m2 starting on day 1 for 46 h. This regimen will be repeated every 14 days. The minimum period of follow-up for each subject will be for 24 months or until death. Study accrual is estimated to require 36 months to complete with each subject followed until 24 months after enrollment of the last subject, or death.

### Registry study

**Fig. 2:**
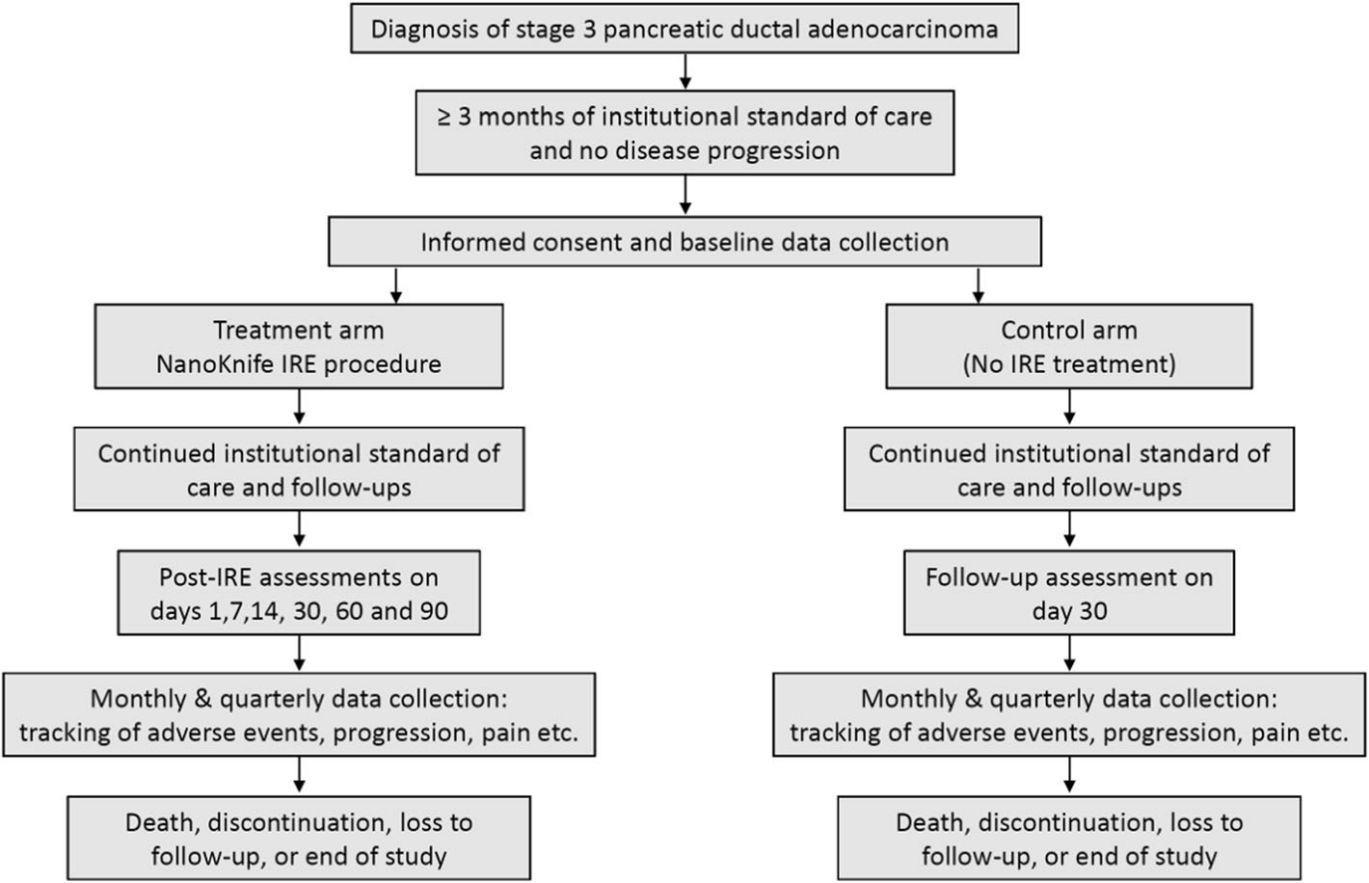
Study design: Registry

The multicenter, observational registry study will include patients with Stage 3 PDAC who received SOC alone and those treated with IRE in addition to SOC (Fig. 2). Both cohorts will be required to undergo at least 3 months of SOC without progression prior to enrollment in the registry. The accrual time for the registry study is 24 months, and each patient will be followed-up for at least 3 months after the study enrolls 266 patients in each cohort. The Registry study will enroll both control and IRE patients from sites where patients are routinely treated with ablation using the NanoKnife System. Additional control patients will also be enrolled from sites that do not offer treatment with the NanoKnife System. The non-NanoKnife sites will be selected to be comparable to the NanoKnife sites with respect to type of center, geography, size, volume of patients with pancreatic cancer, volume of pancreatic cancer surgeries, type of chemotherapy, and duration of chemotherapy.

#### Study objectives

The primary objectives for both the RCT and registry study are to test the hypothesis that IRE with the NanoKnife System improves OS in subjects with Stage 3 PDAC and to assess the safety of IRE compared to the control arm for the RCT or SOC cohort for the registry study. Secondary objectives include comparing PFS, cancer-related pain and quality of life (QOL) for subjects in the control arm versus the IRE arm and to assess procedure-related pain in subjects in the IRE arm.

#### Key eligibility criteria

The RCT will be limited to subjects with cytologically or pathologically confirmed pancreatic adenocarcinoma that are unresectable and who meet the study’s inclusion/exclusion criteria (Table 1). As compared to the stringent eligibility requirements of the RCT, the registry study will have broader inclusion/exclusion criteria allowing for evaluation of real-world evidence regarding the safety and effectiveness of NanoKnife IRE. Patients who have received a wide variety of prior treatments (chemotherapy, chemoradiation or other procedures) as well as those who choose to receive the IRE treatment rather than get randomized are eligible to participate in the registry study. Subjects must have no evidence of disease progression after completion of the 3 months of induction therapy in order to participate in the RCT and registry. Response Evaluation Criteria In Solid Tumours 1.1 (RECIST 1.1) and pancreatic cancer specific secondary endpoints may be used to assess radiologic and clinical outcomes associated with disease progression. Blinded reads of imaging will be performed in a central location by external blinded readers for both the randomized controlled trial and the registry study.

**Table 1:** Patient Inclusion and Exclusion Criteria

Key Inclusion Criteria	
*Randomized Controlled Multicenter Study* ● Signed and dated informed consent form. ● 18 years of age and older. ● Diagnosis of unresectable Stage 3 pancreatic adenocarcinoma cancer cytologically or pathologically confirmed per American Joint Committee on Cancer (AJCC) staging criteria. ● Tumor evaluated as Stage 3 according to National Comprehensive Cancer Network (NCCN) guidelines, based on radiographic imaging or exploratory surgery. ● Maximum axial and anterior to posterior tumor dimension of ≤3.5 cm, after receiving three months of treatment with the mFOLFIRINOX regimen. ● Has received 3 months of treatment with the mFOLFIRINOX regimen. ● Has an Eastern Cooperative Oncology Group (ECOG) performance status of 0 or 1. ● Has an American Society of Anesthesiologists (ASA) classification of physical health status of 1, 2, 3 or 4.	*Multicenter Registry* ● Signed and dated informed consent form ● 18 years of age and older ● Diagnosis of Stage 3 PC cytologically or pathologically confirmed per American Joint Committee on Cancer (AJCC) staging criteria. ● Tumor evaluated as Stage 3 according to National Comprehensive Cancer Network (NCCN) guidelines, based on radiographic imaging or exploratory surgery ● Maximum axial and anterior to posterior tumor dimension of ≤3.5 cm after standard of care. ● Has received 3 months of standard of care per each participating institution’s guidelines ● Has an Eastern Cooperative Oncology Group (ECOG) performance status of 0 or 1. ● Has an American Society of Anesthesiologists (ASA) classification of physical health status of 1, 2, 3 or 4. ● Are at an IRE site and are deemed eligible for IRE and receive ablation using the NanoKnife System. ● Shows no evidence of disease progression based on NCCN guidelines after completing 3 months of standard of care.
Key Exclusion Criteria	
*Randomized Controlled Multicenter Study* ● Subjects who are or may be pregnant as determined by a positive pregnancy test or breastfeeding or male or female patients of reproductive potential who are not willing to employ highly effective birth control from screening to 6 months after the last dose of chemotherapy. ● Unable to tolerate general anesthetic with full skeletal muscle blockade. ● Is actively bleeding, anticoagulated, coagulopathy, or has any of the following hematology results: hemoglobin less than10 g/dL without the support of growth factors or transfusions; absolute neutrophil count less than 1500 cells/mL; or platelet count less than 100,000. ● Has an implanted cardiac pacemaker, defibrillator, electronic device(s) or implanted device(s) with metal parts in the thoracic cavity at the time of IRE. ● Has a history of epilepsy or other neurological diseases. ● Has renal, cardiac, liver, or hematological abnormalities of concern to the investigator. ● Has Stage 3, 4, or 5 chronic kidney disease. ● Is receiving IRE for margin accentuation. ● Has evidence of disease progression at 3 months after FOLFIRINOX treatment. ● Participating in an interventional trial for pancreatic cancer during the study data collection period. ● Did not meet study defined criteria for adequacy of induction treatment at the end of the 3 months.	*Multicenter Registry* ● Participating in an interventional trial for pancreatic cancer during the study data collection period. ● Pregnant or lactating patients or male or female patients of reproductive potential who are not willing to employ highly effective birth control from screening to 6 months after the last dose of chemotherapy ● Unable to tolerate general anesthetic with full skeletal muscle blockade. ● Has an implanted cardiac pacemaker, defibrillator, electronic device(s) or implanted device(s) with metal parts in the thoracic cavity at the time of IRE.

#### Study endpoints and assessments

The primary efficacy endpoint for both the RCT and the registry study will be OS defined as the time (in months) from randomization or enrollment in the registry to the date of death for any reason. Co-primary safety endpoints include time (in months) from randomization or enrollment in the registry to death or new onset of Grade 4 AE, and the development of high-grade complications defined as any AE or SAE with a CTCAE v5.0 grade of 3 or higher.

Secondary endpoints for both studies include a) PFS defined as the time (in months) from randomization or enrollment in the registry to the date of first observed disease progression (per clinical and radiologic criteria), or death from any cause, if death occurs without documented disease progression, b) procedure-related pain in subjects in the IRE arm, as measured by the Brief Pain Inventory (BPI-SHORT FORM) on the day of the procedure and post-procedure days 1, 7, 14 and 30, c) cancer-related pain assessed in the control and IRE arms using the BPI-SHORT FORM at baseline, 3 months, 6 months and at 6-month intervals thereafter, and d) QOL assessed in the control and IRE arms using the EQ-5D questionnaire at baseline, 3 months, 6 months and then at 6-month intervals for the duration of the study.

The registry study will also assess several exploratory endpoints including chemotherapy-free days (after 3 months of SOC), the need for opioids for cancer-related pain, OS from date of pancreatic cancer diagnosis, PFS from date of enrollment, and identification of risk factors and biomarkers associated with outcomes. Additional QOL exploratory endpoints which will be collected include rates of avoidable events associate with chemotherapy in the IRE arm during chemotherapy free days compared to the control arm, unplanned readmissions within 30 days following discharge, and in-hospital mortality.

#### Sample size and statistical considerations

### Randomized controlled trial

Randomization will be conducted centrally. Concurrent randomization to either the IRE or control arm will take place at the completion of 3-month modified FOLFIRINOX administration, at participating sites, and be stratified by the response following 3 months of treatment with mFOLFIRINOX (responder vs. stable disease), planned type of IRE procedure (open or percutaneous) and planned post-procedure treatment (chemotherapy versus radiotherapy/ chemotherapy versus observation). A total of 444 Intent-to-treat population (ITT) subjects (with 222 subjects in each arm) are required for at least 80% power for the primary efficacy endpoint (OS), with the expectation that there will be a total of 380 events in both arms. With the consideration of up to 16% attrition rate in both arms, and assuming a median survival of 12 months and exponential survival, up to 528 subjects (with 264 subjects per arm) will need to be randomized.

The sample size consideration for the primary endpoint OS was based on the assumptions that 1) there will be a 1:1 randomization scheme, 2) median survival will be 12 months and 16 months in the control and the IRE arm, respectively, i.e., a hazard ratio of 0.75 for IRE versus control 3) there will be a 5% 2-sided type I error, 4) there will be an accrual period of 36 months and follow-up period of 24 months after the study is fully enrolled. In addition, based on an assumption that median times to PFS will be 6 months and 8 months in the control and IRE arm, respectively, the study has at least 80% power for analysis of the difference in the secondary endpoint of time to PFS between the IRE and control arms.

### Registry study

The registry study will enroll 266 patients in the IRE cohort and 266 patients in the SOC cohort in order to achieve 90% power for analyzing both the OS and PFS endpoints. The power calculation assumes that it will take 24 months for accrual, and all 532 patients will be followed for at least 3 months after completion of accrual. The sample size consideration was based on the assumptions that the median OS time will be 12 months for SOC cohort and 18 months for the IRE cohort and there will be a 0.05 Alpha level (Type I error rate). For the secondary effectiveness endpoint of PFS, 210 patients will be needed per cohort for 90% statistical power assuming a median PFS of 8 months and 12 months for the SOC and IRE cohorts, respectively, or 116 patients per cohort if the median PFS survival is 8 months for the SOC cohort and 14 months for the IRE cohort.

#### Statistical analysis

### Randomized controlled trial

There will be three analysis populations: 1) an Intent-to-treat population (ITT) which includes all subjects randomized with treatment assignment based on randomization (regardless of actual treatment received); 2) a Modified Intent-to-treat population (mITT) which includes randomized subjects in the control arm who did not receive IRE procedures, and those in the IRE arm who have undergone the IRE procedure, excluding subjects with major protocol deviations. Treatment assignment will be based on the randomized treatment. For subjects who are randomized to control arm but later received IRE treatment, the study data only up to the date of IRE will be considered in this analysis population. Those subjects randomized to the IRE arm but did not receive IRE procedures will be excluded from this analysis population; and 3) a Safety Population consisting of all randomized subjects. If subjects randomized to the control arm receive IRE treatment with NanoKnife, they will be included in the IRE safety population. Similarly, if subjects randomized to the IRE arm did not receive IRE treatment, they will be included in the control safety population.

For the primary efficacy endpoint, the null hypothesis for OS is stated as H_0_: S_control_ = S_IRE_ and the alternative hypothesis is H_1_: S_control_ ≠ S_IRE_, where S_control_ is the median OS time for the control arm, and S_IRE_ is the median OS time for the IRE treatment arm.

The null hypothesis states that there is no difference in median OS time post randomization between the IRE arm and the control arm, while the alternative hypothesis states there is. The trial is powered to show the superiority of OS of 4 months in the IRE arm over the control arm. The above null hypothesis will be tested by a 2-sided log rank test at 0.05 significance level in the final analysis. If the null hypothesis is rejected by the two-sided test and the observed median OS time in the IRE is greater than in the control arm, the alternative hypothesis will be established and the superiority of OS in the IRE arm will be statistically proven.

For the secondary efficacy analysis, the null hypothesis for PFS is H_0_: μ_control_ = μ_IRE_ with the alternative hypothesis being H_1_: μ_control_ ≠ μ_IRE_,where μ_control_ is the median PFS time for the control arm, and μ_IRE_ is the median PFS time for the IRE arm. This study is powered to show the superiority of PFS of 2 months in the IRE arm over the control arm. The above null hypothesis will be tested by a 2-sided log rank test at 0.05 significance level only if the result of testing the primary efficacy endpoint is statistically significant. If the above null hypothesis is rejected by the two-sided test and the observed median PFS time in the IRE is greater than in the control arm, the alternative hypothesis will be established and the superiority of the PFS in the IRE arm will be statistically proven.

Analysis of the primary efficacy endpoint and key secondary endpoints will also be performed for the mITT population as supportive analyses. Safety analysis will be performed on the Safety population. To control the overall type I error rate for testing efficacy endpoints, a fixed-sequence testing procedure will be used to test the primary and secondary efficacy endpoints in a predefined order. The test will be performed in sequence and significance of OS endpoint is required in order to proceed to the testing of PFS endpoint. All statistical tests will be at the two-sided 0.05 significance level and the corresponding *p*-values and 95% confidence intervals (CIs) will be reported. Subjects alive or lost to follow-up at the time of analysis will be censored at their last date of follow-up. In the final analysis, OS will be compared between the randomized treatment arms using a two-sided log-rank test at a type I error rate of 0.05. ITT analysis will be the primary analytic methods, supported with mITT and Cox proportional hazards analysis. Kaplan-Meier methods will be used to summarize survival distribution of OS and median OS time for each treatment arm. The relative treatment effect between the IRE arm and the control arm with respect to OS will be estimated by the hazard ratio (HR) and the associated two-sided 95% CIs using the Cox proportional hazards model.

There will be one planned interim analysis with an early stopping rule for adverse events and for superiority associated with the primary efficacy endpoint. Stopping rules for adverse events will include (but will not be limited to) two IRE related ventricular arrhythmias during the IRE procedure that require synchronized cardioversion or defibrillation or one death determined by the investigator and the Data Monitoring Committee (DMC) to be related to IRE.

There will be a single OS-based threshold for stopping the trial for superiority using a pre-specified Haybittle-Peto boundary with an alpha level of 0.001. If the IRE arm demonstrates superiority over the control arm with *P*-value< 0.001 following the interim analysis, the study may be terminated early, depending on the safety profile. Since the study will be powered based on an OS primary endpoint, there will not be an adverse events related threshold for stopping the trial for superiority.

The interim analysis will not be used to lead to early termination of the study because of futility (e.g., the IRE arm does not demonstrate superiority over the control arm). The DMC will have the ability to make recommendations after reviewing the results of the interim analysis or through safety monitoring, and in conjunction with the study sponsor, can decide to continue or terminate the study to save resources.

The interim analysis will be conducted when approximately 251 (66% of the total 380) deaths have been observed in the overall ITT population. As part of the interim analysis, the assumptions about the sample size estimation will also be evaluated. The final analyses of OS will be conducted at the 5% level of significance when approximately 380 death events occurred. Interim data will be evaluated by the independent DMC.

Subjects who do not have disease progression and have not died, will be censored at the date of last tumor assessment. Subjects with a single missing radiologic or clinical assessment immediately prior to a visit with documented disease progression (or death) will be analyzed as a PFS event at the time of the clinical or radiologic assessment that shows progression. Subjects with two or more consecutive missing radiologic or clinical assessments immediately prior to an assessment without documented progression (or death) will be censored at the date of last assessment when the subject was documented as progression-free prior to the first of the two or more missing assessments. PFS will be analyzed using similar statistical methods as that for the OS endpoint.

Pain Severity Scores and average Pain Interference Score will be derived for each subject from BPI-SHORT FORM questionnaire. QOL measures will be derived for each subject from the EQ-5D questionnaire, including the EQ Visual Analogue Scale (VAS) and EQ-5D summary index. Both absolute change and percent change from baseline will be analyzed by treatment arm and by study visit for all of the above.

For the primary safety endpoints, a log-rank test will be used to compare the time (in months) from randomization to death or new onset of Grade 4 AEs for the two treatment arms, with data censored at the time of loss to follow up or study discontinuation. The number and proportion of subjects with a high-grade complication defined as any AEs or SAEs with a CTCAEv5.0 grade of 3 or higher will be summarized by treatment arm and CTCAE grade.

### Registry study

There will be two analysis populations including 1) an Effectiveness Population which consists of all enrolled patients who will be matched by propensity score quintiles, all of whom have received at least 3 months of therapy per SOC without evidence of disease progression, and 2) a Safety Population which consists of all enrolled patients with the cohort assignment based on the actual treatment received.

For the primary efficacy endpoint, the null hypothesis for OS is stated as H_0_: S_soc_ = S_IRE_ and the alternative hypothesis is H_1_: S_soc_ ≠ S_IRE_, where S_soc_ is the median OS time for the SOC cohort, and S_IRE_ is the median overall survival time for the IRE cohort. For the secondary efficacy analysis, the null hypothesis for PFS is H_0_: μ_soc_ = μ_IRE_ with the alternative hypothesis being H_1_: μ_soc_ ≠ μ_IRE_, where μ_soc_ is the median PFS time for the SOC cohort, and μ_IRE_ is the median PFS time for the IRE arm. The null hypothesis for each state that there is no difference in median OS or PFS time post the initial 3 months of therapy between the IRE cohort and the SOC cohort, while the alternative hypothesis states there is. This study is powered to show the superiority of OS and PFS in the IRE cohort over the SOC cohort. The above null hypotheses will be tested by a 2-sided log rank test stratified by propensity score quintiles at the 0.05 significance level. The PFS endpoint will be tested only if the result of testing the primary effectiveness endpoint is statistically significant. Exploratory subgroup analyses will include patient’s sex (male or female), and other factors to be determined after the study team reviews the final analyses of primary and secondary endpoints.

There will be one planned interim analysis for the registry study, to be conducted when 50% of 266 patients in either cohort die, or per the recommendation of the DMC. The purpose of the interim analysis is to examine accrual rate and check the assumptions about the median survival times (i.e., 12 months for SOC cohort and 18 months for the IRE cohort), to ensure that the study will have sufficient power for planned analyses.

Exploratory analyses for the registry study will include OS from date of diagnosis, PFS from date of diagnosis, chemotherapy-free days per 100 person-months of follow-up after enrollment, proportion of patients treated with opioids for cancer-related pain at 3, 6, 12, 18 and 24 months after enrollment, mean Morphine Milligram Equivalent (MME)/Day among patients using opioids in each cohort at these time points, rates of avoidable events associated with chemotherapy in the IRE arm during chemotherapy free days compared to the control arm, 30-day unplanned readmissions, in-hospital mortality and identification of risk factors and biomarkers associated with outcomes. These exploratory analyses will be descriptive and, as deemed necessary, will be stratified by propensity score quintiles, or including propensity scores as a covariate. Unless explicitly stated otherwise in the analysis plan, the exploratory analyses will be based on the Effectiveness population. The analysis method for OS from date of diagnosis and PFS from date of diagnosis will be the same as for OS from date of enrollment. Poisson regression model will be used for analyzing chemotherapy-free days, and logistic regression model will be used for analyzing other binary endpoints.

## Discussion

A diagnosis of Stage 3 PDAC is associated with poor outcomes and a low 5-year survival rate. The present studies are designed to test the hypothesis that IRE treatment with the NanoKnife System when combined with a standard chemotherapy regimen improves overall survival with an acceptable safety profile. Additional goals of the study are to also provide evidence that IRE treatment can also improve PFS, reduce cancer-related pain and improve QOL.

These two studies will be run in parallel to collect both level 1 clinical evidence and real-world data. This will be accomplished via multicenter studies using both a randomized, controlled trial design with more stringent inclusion/exclusion criteria and an observational registry enrolling of individuals which are more reflective of the broader population of patients with Stage 3 PDAC. This dual study approach will be useful towards addressing the inherent limitations associated with using either of these study designs for assessing the treatment of cancer patients [32, 33]. For randomized, controlled trials, a typical limitation is the inability to generalize the results to a larger patient population since the study subjects may not be representative of all cancer patients. While registry studies can provide insights on clinical outcomes associated with newer therapies, they have inherent weaknesses that impact the ability to make comparisons between nonrandomized patient cohorts.

The present studies are designed to contribute to the comprehensive information and clinical evidence reported to date supporting the safety and efficacy associated with IRE treatment in patients with Stage 3 PDAC. Positive results from the study in terms of clinical efficacy with an acceptable safety profile would support the standard use of IRE in patients with Stage 3 PDAC.

## Data Availability

The authors plan to publish the results of these studies at a later date after patient enrollment and follow-up is completed and the associated data is analyzed. Authorship will be handled according to standards set by the International Committee of Medical Journal Editors.

## Abbreviations

AE: Adverse Event
AJCC: American Joint Committee on Cancer
ASA: American Society of Anesthesiologists
BPI: Brief Pain Inventory
CRF: case Report Form
CTCAE: Common Terminology Criteria for Adverse Events
DMC: Data Monitoring Committee
ECOG: Eastern Cooperative Oncology Group
FOLFIRINOX: Regimen of leucovorin, fluorouracil, irinotecan, oxaliplatin
IRB: Institutional Review Board
IRE: Irreversible Electroporation
ITT: Intention-to-Treat
mITT: Modified Intent-to-treat
NCCN: National Comprehensive Cancer Network
NCI: National Cancer Institute
OS: Overall Survival
PDAC: Pancreatic Ductal Adenocarcinoma
PET: Positron Emission Tomography
PFS: Progression-Free Survival
QOL: Quality of Life
RECIST: Response Evaluation Criteria In Solid Tumours
SAE: Serious Adverse Event
SD: Standard Deviation
SOC: Standard of Care
TTP: Time to Progression

## References

[1] National Cancer Institute, Surveillance, Epidemiology, and End Results Program. CancerStat Facts: Pancreatic Cancer. https://seer.cancer.gov/statfacts/html/pancreas.html. Accessed 6 Jan 2020.

[2] van Roessel S, Kasumova GG, Verheij J, Najarian RM, Maggino L, de Pastena M, Malleo G, Marchegiani G, Salvia R, Ng SC, de Geus SW, Lof S, Giovinazzo F, van Dam JL, Kent TS, Busch OR, van Eijck CH, Koerkamp BG, Abu Hilal M, Bassi C, Tseng JF, Besselink MG (2018). International validation of the eighth edition of the American joint committee on Cancer (AJCC) TNM staging system in patients with resected pancreatic cancer. JAMA Surg.

[3] Loehrer PJ, Feng Y, Cardenes H, Wagner L, Brell JM, Cella D, Flynn P, Ramanathan RK, Crane CH, Alberts SR, Benson AB (2011). Gemcitabine alone versus gemcitabine plus radiotherapy in patients with locally advanced pancreatic cancer: an eastern cooperative oncology group trial. J Clin Oncol.

[4] Allen PJ, Kuk D, Castillo CF, Basturk O, Wolfgang CL, Cameron JL, Lillemoe KD, Ferrone CR, Morales-Oyarvide V, He J, Weiss MJ, Hruban RH, Gönen M, Klimstra DS, Mino-Kenudson M (2017). Multi-institutional validation study of the American joint commission on Cancer (8th edition) changes for T and N staging in patients with pancreatic adenocarcinoma. Ann Surg.

[5] Conroy T, Desseigne F, Ychou M, Bouché O, Guimbaud R, Bécouarn Y, Adenis A, Raoul JL, Gourgou-Bourgade S, de la Fouchardière C, Bennouna J, Bachet JB, Khemissa-Akouz F, Péré-Vergé D, Delbaldo C, Assenat E, Chauffert B, Michel P, Montoto-Grillot C, Ducreux M (2011). FOLFIRINOX versus gemcitabine for metastatic pancreatic cancer. N Engl J Med.

[6] Blazer M, Wu C, Goldberg RM, Phillips G, Schmidt C, Muscarella P, Wuthrick E, Williams TM, Reardon J, Christopher Ellison E, Bloomston M, Bekaii-Saab T (2015). Neoadjuvant modified (m) FOLFIRINOX for locally advanced unresectable (LAPC) and borderline resectable (BRPC) adenocarcinoma of the pancreas. Ann Surg Oncol.

[7] Tempero MA, Malafa MP, Chiorean EG, Czito B, Scaife C, Narang AK, Fountzilas C, Wolpin BM, al-Hawary M, Asbun H, Behrman SW, Benson AB, Binder E, Cardin DB, Cha C, Chung V, Dillhoff M, Dotan E, Ferrone CR, Fisher G, Hardacre J, Hawkins WG, Ko AH, LoConte N, Lowy AM, Moravek C, Nakakura EK, O’Reilly EM, Obando J, Reddy S, Thayer S, Wolff RA, Burns JL, Zuccarino-Catania G (2019). Pancreatic Adenocarcinoma, Version 1.2019. J Natl Compr Cancer Netw.

[8] Hammel P, Huguet F, van Laethem JL, Goldstein D, Glimelius B, Artru P, Borbath I, Bouché O, Shannon J, André T, Mineur L, Chibaudel B, Bonnetain F, Louvet C, LAP07 Trial Group (2016). Effect of chemoradiotherapy vs chemotherapy on survival in patients with locally advanced pancreatic cancer controlled after 4 months of gemcitabine with or without erlotinib: the LAP07 randomized clinical trial. JAMA..

[9] Davalos RV, Mir IL, Rubinsky B (2005). Tissue ablation with irreversible electroporation. Ann Biomed Eng.

[10] Bower M, Sherwood L, Li Y (2011). Irreversible electroporation of the pancreas: definitive local therapy without systemic effects. J Surg Oncol.

[11] Choi JW, Lu DS, Osuagwu F, Raman S, Lassman C (2014). Assessment of chronological effects of irreversible electroporation on hilar bile ducts in a porcine model. Cardiovasc Intervent Radiol.

[12] Narayanan G, Bhatia S, Echenique A, Suthar R, Barbery K, Yrizarry J (2014). Vessel patency post irreversible electroporation. Cardiovasc Intervent Radiol.

[13] Phillips M, Maor E, Rubinsky B (2010). Nonthermal irreversible electroporation for tissue decellularization. J Biomech Eng.

[14] Lafranceschina S, Brunetti O, Delvecchio A, Conticchio M, Ammendola M, Currò G (2019). Systematic review of irreversible electroporation role in management of locally advanced pancreatic cancer. Cancers (Basel).

[15] Ansari D, Kristoffersson S, Andersson R, Bergenfeldt M (2017). The role of irreversible electroporation (IRE) for locally advanced pancreatic cancer: a systematic review of safety and efficacy. Scand J Gastroenterol.

[16] Moris D, Machairas N, Tsilimigras DI, Prodromidou A, Ejaz A, Weiss M, Hasemaki N, Felekouras E, Pawlik TM (2019). Systematic review of surgical and percutaneous irreversible electroporation in the treatment of locally advanced pancreatic cancer. Ann Surg Oncol.

[17] Belfiore G, Belfiore MP, Reginelli A, Capasso R, Romano F, Ianniello GP, Cappabianca S, Brunese L (2017). Concurrent chemotherapy alone versus irreversible electroporation followed by chemotherapy on survival in patients with locally advanced pancreatic cancer. Med Oncol.

[18] Månsson C, Brahmstaedt R, Nilsson A, Nygren P, Karlson BM (2016). Percutaneous irreversible electroporation for treatment of locally advanced pancreatic cancer following chemotherapy or radiochemotherapy. Eur J Surg Oncol.

[19] Martin RC, McFarland K, Ellis S, Velanovich V (2012). Irreversible electroporation therapy in the management of locally advanced pancreatic adenocarcinoma. J Am Coll Surg.

[20] Martin RC, McFarland K, Ellis S, Velanovich V (2013). Irreversible electroporation in locally advanced pancreatic cancer: potential improved overall survival. Ann Surg Oncol.

[21] Martin RC, Kwon D, Chalikonda S, Sellers M, Kotz E, Scoggins C (2015). Treatment of 200 locally advanced (stage III) pancreatic adenocarcinoma patients with irreversible electroporation: safety and efficacy. Ann Surg.

[22] Paiella S, Butturini G, Frigerio I, Salvia R, Armatura G, Bacchion M, Fontana M, D''Onofrio M, Martone E, Bassi C (2015). Safety and feasibility of irreversible electroporation (IRE) in patients with locally advanced pancreatic cancer: results of a prospective study. Dig Surg.

[23] Vogel JA, Rombouts SJ, de Rooij T, van Delden OM, Dijkgraaf MG, van Gulik TM, van Hooft JE, van Laarhoven HW, Martin RC, Schoorlemmer A, Wilmink JW, van Lienden KP, Busch OR, Besselink MG (2017). Induction chemotherapy followed by resection or irreversible electroporation in locally advanced pancreatic cancer (IMPALA): a prospective cohort study. Ann Surg Oncol.

[24] Scheffer HJ, Vroomen LG, de Jong MC, Melenhorst MC, Zonderhuis BM, Daams F (2017). Ablation of locally advanced pancreatic cancer with percutaneous irreversible electroporation: results of the phase I/II PANFIRE study. Radiology..

[25] Ruarus AH, Vroomen LGPH, Geboers B, van Veldhuisen E, Puijk RS, Nieuwenhuizen S, Besselink MG, Zonderhuis BM, Kazemier G, de Gruijl TD, van Lienden KP, de Vries JJJ, Scheffer HJ, Meijerink MR (2020). Percutaneous irreversible electroporation in locally advanced and recurrent pancreatic cancer (PANFIRE-2): a multicenter, prospective, single-arm, Phase II study. Radiology.

[26] Narayanan G, Hosein PJ, Beulaygue IC, Froud T, Scheffer HJ, Venkat SR, Echenique AM, Hevert EC, Livingstone AS, Rocha-Lima CM, Merchan JR, Levi JU, Yrizarry JM, Lencioni R (2017). Percutaneous image-guided irreversible electroporation for the treatment of unresectable, locally advanced pancreatic adenocarcinoma. J Vasc Interv Radiol.

[27] Sugimoto K, Moriyasu F, Tsuchiya T, Nagakawa Y, Hosokawa Y, Saito K, Tsuchida A, Itoi T (2018). Irreversible electroporation for nonthermal tumor ablation in patients with locally advanced pancreatic cancer: initial clinical experience in Japan. Intern Med.

[28] Lambert L, Horejs J, Krska Z, Hoskovec D, Petruzelka L, Krechler T, Kriz P, Briza J (2016). Treatment of locally advanced pancreatic cancer by percutaneous and intraoperative irreversible electroporation: general hospital cancer center experience. Neoplasma..

[29] Kluger MD, Epelboym I, Schrope BA, Mahendraraj K, Hecht EM, Susman J, Weintraub JL, Chabot JA (2016). Single-institution experience with irreversible electroporation for T4 pancreatic cancer: first 50 patients. Ann Surg Oncol.

[30] Leen E, Picard J, Stebbing J, Abel M, Dhillon T, Wasan H (2018). Percutaneous irreversible electroporation with systemic treatment for locally advanced pancreatic adenocarcinoma. J Gastrointest Oncol.

[31] Holland MM, Bhutiani N, Kruse EJ, Weiss MJ, Christein JD, White RR, Huang KW, Martin RCG (2019). A prospective, multi-institution assessment of irreversible electroporation for treatment of locally advanced pancreatic adenocarcinoma: initial outcomes from the AHPBA pancreatic registry. HPB (Oxford).

[32] Foroughi S, Wong HL, Gately L, Lee M, Simons K, Tie J, Burgess AW, Gibbs P (2018). Re-inventing the randomized controlled trial in medical oncology: the registry-based trial. Asia Pac J Clin Oncol.

[33] Booth CM, Karim S, Mackillop WJ (2019). Real-world data: towards achieving the achievable in cancer care. Nat Rev Clin Oncol.

